# Dietary Changes and Its Impact on Quality of Life among Malay Breast and Gynaecological Cancer Survivors in Malaysia

**DOI:** 10.31557/APJCP.2020.21.12.3689

**Published:** 2020-12

**Authors:** Nadzirah Hanis Zainordin, Ruzita Abd Talib, Mohd Razif Shahril, Suhaina Sulaiman, Norimah A Karim

**Affiliations:** 1 *Nutritional Sciences Programme, Center for Community Health Studies (ReaCH), Faculty of Health Sciences, Universiti Kebangsaan Malaysia, Jalan Raja Muda Abdul Aziz, 50300 Kuala Lumpur, Malaysia. *; 2 *School of Nutrition and Dietetics, Faculty of Health Sciences, Universiti Sultan Zainal Abidin, Gong Badak Campus, 21300 Kuala Nerus, Terengganu, Malaysia. *; 3 *Dietetics Programme, Centre for Healthy Aging and Wellness (H-Care), School of Healthcare Sciences, Faculty of Health Sciences, Universiti Kebangsaan Malaysia, Jalan Raja Muda Abdul Aziz, 50300 Kuala Lumpur, Malaysia. *

**Keywords:** Quality of life, dietary intake, breast cancer, gynaecological cancer, cancer survivors

## Abstract

**Objective:**

Fear of cancer recurrent, side effects of treatment and belief in food taboos encourage cancer survivors to make changes in their dietary practices after diagnosis of cancer. The objective of this study was to determine the impact of dietary changes on quality of life (QoL) among Malay breast and gynaecological cancer survivors.

**Methods:**

Questionnaire of dietary changes was modified from WHEL study and adapted to typical Malay’s food intake in Malaysia. A total of 23 items were listed and categorized by types of food and cooking methods. Four categories of changes “increased”, “decreased”, “no changes” or “stopped” were used to determine the changes in dietary practices. Score one (+1) is given to positive changes by reference to WCRF/AICR and Malaysia Dietary Guideline healthy eating recommendations. Malay EORTC QLQ-C30 were used to determine the QoL. Sociodemographic, clinical characteristics and anthropometric measurement were also collected.

**Results:**

The mean age of the subjects (n=77) was 50.7±7.8 years old with duration of survivorship 4.0±3.1 years. Subjects mean BMI was 27.8±4.9 kg/m^2^ which indicate subjects were 31.2% overweight and 32.5% obese. The percentage score of positive dietary changes was 34.7±16.4%. Positive dietary changes were increased intake of green leafy vegetable (49.4%), cruciferous vegetable (46.8%) and boiling cooking methods (45.5%). Subjects reduced their intake of red meat (42.9%), sugar (53.2%) and fried cooking method (44.2%). Subjects stopped consuming milk (41.6%), c 2008-5862 heese (33.8%) and sweetened condensed milk (33.8%). With increasing positive dietary changes, there was a significant improvement on emotional function (rs=0.27; p=0.016) and reduced fatigue symptoms (rs=-0.24; p=0.033).

**Conclusion:**

Positive changes in dietary intake improved emotional function and reduced fatigue symptoms after cancer treatment. By knowing the trend of food changes after cancer treatment, enables the formation of healthy food intervention implemented more effective.

## Introduction

The advancement in early detection and treatment of cancer has improved the survival rate in cancer patients (Miller et al., 2019). In the United States, cancer survivors are expected to increase from 16.9 million in 2019 to 22.1 million in 2030 (Miller et al., 2019). In Malaysia, the first population-based cancer survival project carried out by the Malaysia Study on Cancer Survival (MySCan) recorded 72,884 cases from 2007 to 2011. Of these cases, 35.7% of the cancer patients successfully lived through more than 5 years since diagnosis (National Cancer Registry [NCR], 2018). According to the MySCan, the survival rate above 5 years for breast cancer was 66.8%, cervical cancer was 51.6%, ovarian cancer was 54.5% and uterine corpus cancer was 70.6% (NCR, 2018). Among the ethnic groups in Malaysia, the survival rate was lowest among the Malays compared to the Chinese and Indian (NCR, 2018). Bhoo-Pathy et al., (2012) reported the lower survival rate among the Malays population was attributed to the late diagnosis and non-compliance to treatment. Furthermore, the practices of cultural food abstinence or locally knows as “pantang-larang”, among the Malays cancer patients may lead to a lower quality of life compared to the Chinese ethnic (Yusuf et al., 2013).

After cancer treatment, breast and gynaecological cancer patients will experience some forms of short- or long-term side effects such as fatigue, pain, lymphedema, cognitive dysfunction, osteoporosis, stress, menopausal symptoms, sexuality and fertility issues after the treatment (Agrawal, 2014; Campbell et al., 2019). These side effects can impact negatively on the patient’s QoL (Akhtari-Zavare et al., 2018). However, studies had shown that through healthy eating practices and better nutritional status, it could improve or reduce some of these side effects and therefore improve QoL of the cancer survivors (Lis et al., 2012; Kim et al., 2018). 

Cancer patients were highly motivated to change their dietary intake to improve the wellbeing and prevent cancer recurrence (Beenken et al., 2016). Nurses’ Health Study (Izano et al., 2013), Life After Cancer Epidemiology Study (Kwan et al., 2009), Health, Eating, Activity, and Lifestyle (George et al., 2011) and Women Intervention Nutrition Study (Thomson et al., 2014) have shown that changes in food intake pattern have the potential in affecting survivorship. The World Cancer Research Fund/American Institute [WCRF/AICR] (2018a) had concluded that increased intake in whole grains, vegetables, fruits and beans and reduced intake in fast food, processed food, red meat, sweetened drinks and alcohol can reduce the risk of cancer. However, the effects of cancer treatment may alter the metabolism, physiological and psychological aspects of the patient which can impact negatively on the quality of their nutritional status (Lis et al., 2012). Therefore, it is also recommended by the WCRF/AICR (2018a) that cancer patients shall maintain healthy body weight, be physically active, and do not rely on supplements to complement the dietary intake in cancer prevention.

In Malaysia, studies conducted by Shaharudin et al., (2013) and Yaw et al., (2014) reported that cancer survivors had changed their food intake by reducing the intake of red meat, noodles, seafood, poultry, high-fat and sugary foods. Cancer survivors have increased the intake of fish, fruits, vegetables and whole grains. The reasons for these changes were attributed by doctors’ and dietitians’ advice, the recommendation from other cancer survivors as well as the desire to recover from cancer (Shaharudin et al., 2013). Despite changing the food intake after cancer treatment, it was found that cancer survivors did not achieve the recommended healthy food intake (Zainuddin et al., 2017). Zainuddin et al., (2017) showed that cancer survivors did not achieve recommendation for intake of fibre, monounsaturated fatty acids, polyunsaturated fatty acids, calcium, iron and iron as recommended by the Malaysian Recommended Nutrient Intake (RNI) (National Coordinating Committee on Food and Nutrition [NCCFN],2017). This indicated that although cancer survivors made dietary changes, they have not improved their diet quality.

Most of the studies on cancer survivors in Malaysia were focused on the aspects of food intake status, quality of food intake and changes in food intake among cancer survivors (Shaharudin et al., 2013; Yaw et al., 2014; Zainuddin et al., 2017). The explorations of the association between dietary changes and QoL among cancer survivors were limited. Since different ethnic groups may have different patterns or beliefs in their food intake (Yusuf et al., 2013) and the survival rate had improved among cancer patients (Miller et al., 2019), therefore this study aimed to explore the impact of dietary changes on QoL among the Malays breast and gynaecological cancer survivors with longer survivorship. 

## Materials and Methods

This cross-sectional study was approved by the Medical Research Ethics Committee MREC (NMRR-15-1435-26831) and UKM Ethics Committee (NN-049-2015). Patients who attended the follow-up appointment at the Radiotherapy and Oncology and the Gynae-oncology outpatient clinic of Kuala Lumpur Hospital (HKL) and Hospital Canselor Tuanku Muhriz UKM (HCTM) as referral hospitals in the Central Region of Malaysia were recruited as the subjects. Convenience sampling was used in this study. 

Subjects were recruited based on the following inclusion criteria: Malaysian Malay aged between 18 to 65 years old; confirmed diagnose of breast or gynaecological cancer (cervical, ovarian, uterine, vaginal and vulvar); cancer staging of I, II or III; completed all primary clinical treatment e.g. surgery and/or chemotherapy and/or radiotherapy for more than 6 months and no history of cancer recurrence. All prospective subjects’ medical records were reviewed. The potential subject’s name was called up by public announcement in the clinic and informed about the study. Written informed consent was obtained from the subjects who agreed to participate before the study began. Subjects were excluded if they could not speak, communicate and understand Malay language. Only subjects had made dietary changes since cancer diagnosis were included in this study. 

The subjects’ socio-demographic, dietary changes and QoL were obtained via a self-reported questionnaire. Digital weighing scales (SECA Model 880, Germany) was used to measure body weight to the nearest 0.1 kg using. Height was measured to the nearest 0.1 cm using a SECA Portable stadiometer (Model 213, Germany). Body mass index (BMI) calculated and distribution categorial based on WHO (1995). 

*Dietary Changes Questionnaire*

The dietary changes questionnaire was adapted and modification from Women’ Healthy Eating and Living Study (WHEL) (Thomson et al., 2002) and Shaharudin et al., (2013) study. The questionnaire had a total list of 23 items. Seventeen (17) of these items were related to food and six were related to cooking methods. The food items chosen were shortlisted from a list of food based on a preliminary study of 24-hour diet recall by 70 cancer survivors. A Malaysian recipe books was also referred to identify ingredients and cooking methods used (Ismail 2015; Wan 2014). Through this process, items were shortlisted according to the recommendations of healthy eating by WCRF/AICR (2018a) and Malaysian Dietary Guideline (MDG) (NCCFN 2010). The purpose was to ensure that the selected items were based on scientific evidence on cancer and healthy intake. The subjects checked on foods that were consumed before diagnosis, whereas responses on changes after diagnosis was classified as either “increased”, “decreased”, “no changes” or “stopped”. An open-ended question was also included in the questionnaire to explore further the subjects’ reasons in practicing the dietary changes. 

Changes were considered as positive if there were increased intake of fruits, vegetables, fish, milk, cheese, legumes, soy and beans. Positive scores were also given if there was decreased or no intake of red meat, sugar, sweetened condensed milk, salt, seasoning and coconut milk. Additionally, if there were an increased in low-fat cooking method e.g. using stir-frying, grilling, stimming and boiling it would be considered positive changes as well. A positive change was allocated with 1 point (+1) whereas negative changes were given zero scores (0). A higher score indicated positive dietary change after the participants were diagnosed with cancer. 

*Quality of life*

Quality of life (QoL) was assessed using the EORTC QLQ-C30 module developed by European Organization or Research and Treatment of Cancer (Fayers et al., 2001). This questionnaire had been validated in the Malay language by Yusoff, Low and Yip (2010). The EORTC QLQ-C30 consisted of 30 multi-dimensional cancer-specific questions developed to assess the QoL of cancer patients. The answers to this questionnaire were based on the past one-week experiences. The questionnaire was divided into three scales: functional scale, symptoms scale and global health scale (GHS). The functional scale and symptom scale items were rated on a four-point Likert scales with 1 being “not at all” to 4 beings “very much”. Item Q29 and Q30 of GHS are rated with a seven-level Likert scale from 1 being “very poor” to 7 being “excellent”. The raw score for each subscale was linearly transformed to standardize score in the range 0 to 100 according to the EORTC QLQ-C30 scoring manual guideline (Fayers et al., 2001). A higher score indicates better QoL for functioning and GHS scale but more severe in the symptomatic problems. 

*Statistical Analysis*

Descriptive statistics were used to summarize sociodemographic, clinical characteristics, anthropometric measurements, dietary changes and QoL. Independence t-test, Mann-Whiney U test or Chi-Square was performed to determine whether any differences in the breast or gynaecological cancer across sociodemographic, clinical characteristics, anthropometric measurements and QoL. Differences were defined to be significant when p-value is less than 0.05. The associations between dietary changes score and QoL were analysed using Spearman’s Rho. All statistical analysis were carried out using IBM SPSS Statistics for Windows, version 22 (IBM Corp., Armonk, N.Y., USA). 

## Results

A total of 77 breast (n=56) and gynaecological (n=21) cancer survivors participated in the survey. The mean age was 50.7±7.8 years old. Most of the subjects were married (72.7%), had a secondary education level (51.9%) and were employed (53.2%). Clinical characteristics of breast cancer subjects indicate diagnosis cancer with of stage II (51.8%), mean survivorships 3.8±2.9 years and undergone all three modalities of clinical treatments; surgery, radiotherapy and chemotherapy (73.2%). Whereas, gynaecological cancer subjects were presented with ovarian cancer (57.1%), at stage I (66.7%), mean survivorship of 4.7±3.5 years and went through both surgery and chemotherapy treatment (53.4%). Mean body mass index (BMI) was 27.8±4.9kg/m2. Among the breast and gynaecological cancer subjects, a significant difference was observed in the cancer staging (p<0.001) and type of treatment (p<0.001) (Table 1). 

Table 2 demonstrates the types of foods consumed before and after the diagnoses of cancer. The mean score of dietary changes were 34.7±16.4% (Range 4.35-69.6). Subjects changed intake by increased their intake of green leafy vegetable (49.4%), cruciferous vegetable (46.8%) and reduced intake of red meat (42.9%) and sugar (53.2%). Subjects stopped consuming milk (41.6%), cheese (33.8%) and sweetened condensed milk (33.8%). In terms of cooking methods, the subjects had reduced frying (44.2%) and increased boiling (45.5%) cooking methods in their food preparation. The three main reasons in practicing the dietary changes by the subjects were fear of cancer recurrence, treatment side effects and risk in developing non-communicable diseases (Table 3). 

Table 4 illustrates the QoL among the cancer survivors and their correlation with the dietary changes score. When comparing between the breast and gynaecological subjects, the breast cancer subjects presented with a significantly higher symptom of nausea and vomiting (p=0.024) and pain (p=0.024). A significant correlation was observed in the emotional functioning scale (rs=0.27; p=0.024) and fatigue scale (rs=-0.24; p=0.017) with the dietary changes score among all the cancer subjects. This indicates that positive changes in food intake after diagnosis of cancer, will increase emotional function and reduce fatigue symptoms. 

**Table 1:** Sociodemographic and Clinical Characteristics of the Subject (n=77)

Characteristics	All cancer n=77	Breast CA (n=56)	Gynaecological CA (n=21)	p-value
Age (years)				
Mean ± SD	50.7± 7.8	51.3±6.7	49.1±9.9	0.258
Marital status				0.544
Single/ Divorced/Widowed	21 (27.3)	15 (26.8)	6 (28.6)	
Married	56 (72.7)	41 (73.2)	15 (71.4)	
Education				0.617
Secondary and below	40 (51.9)	28 (50.0)	12 (57.1)	
Tertiary and above	37 (48.1)	28 (50.0)	9 (42.9)	
Employment status				
Employed	41 (53.2)	33 (58.9)	8 (38.1)	0.128
Unemployed/retired	36 (46.8)	23(41.1)	13 (61.9)	
Monthly household income (RM)				
<RM 1,500	15 (19.5)	10 (17.9)	5 (23.8)	0.595
RM1500.00-RM 2999.00	13 (16.9)	10 (17.9)	3 (14.3)	
RM3000.00- RM4999.00	23 (29.9)	15 (26.8)	8 (38.1)	
>RM5000.00	26 (33.8)	21 (37.5)	5 (23.8)	
Stage of cancer				
Stage I	25 (32.5)	11 (19.6)	14 (66.7)	<0.001*
Stage II	34 (44.2)	29 (51.8)	5 (23.8)	
Stage III	18 (23.4)	16 (28.6)	2 (9.5)	
Types of gyne-cancer				
Ovarian	12 (57.1)	-	12 (57.1)	
Uterine	3 (14.3)	-	3 (14.3)	
Cervical/virginal/vulvar	6 (28.6)	-	6 (28.6)	
Duration of survivorship (years)				
Mean ± SD	4.0±3.1	3.8±2.9	4.7±3.5	0.284
Type of treatment				
Surgery	10 (13.0)	4 (7.1)	6 (28.6)	<0.001*
Surgery+radiotherapy	5 (6.5)	4 (7.1)	1 (4.8)	
Surgery+chemotherapy	18 (23.4)	7 (12.5)	11 (52.4)	
Radiotherapy+Chemotherapy	2 (2.6)	0 (0)	2 (9.5)	
Surgery+Radiotherap+Chemotherapy	42 (54.5)	41 (73.2)	1 (4.8)	
Anthropometric				
Height (cm)	153.9±4.7	153.6±4.7	154.7±4.8	0.384
Weight (kg)	65.8±11.8	64.7±10.6	68.5±14.3	0.208
Body mass index (kg/m^2^)	27.8±4.9	27.4±5.3	28.6±5.9	0.32
Normal	28 (36.4)	21 (37.5)	7 (33.3)	0.812
Overweight	24 (31.2)	18 (32.1)	6 (28.6)	
Obese	25 (32.5)	17 (30.3)	8 (38.0)	

*p<0.05 significant using the Chi-square test

**Table 2:** Changes in Food and Cooking Methods after Cancer Diagnosis (n=77)

Food /cooking methods	Pre-diagnosis consumption n (%)	Changes after cancer diagnosis (n=77)			
		Increase	Decreased	No Changes	Stop consumed
Mean score	34.7±16.4% (Range 4.35-69.6)				
Food					
Fish^a^	77 (100)	20 (26.0)	14 (18.2)	40 (51.9)	3 (3.9)
Red meat^b^	77 (100)	1 (1.3)	33 (42.9)	12 (15.6)	31 (40.3)
Beans and lentils^a^	72 (93.5)	4 (5.2)	16 (20.8)	48 (62.3)	9 (11.7)
Soy products^a^	62 (80.5)	3 (3.9)	19 (23.2)	34 (44.2)	21 (27.3)
Tropical fruits^a^	77 (100)	29 (37.7)	6 (7.8)	41 (53.2)	1 (1.3)
Non-tropikal fruits^a^	77 (100)	27 (31.2)	9 (11.7)	44 (57.1)	0 (0)
Legumes^a^	76 (98.7)	18 (23.4)	13 (16.9)	44 (57.1)	2 (2.6)
Green leafy vegetable^a^	77 (100)	38 (49.4)	4 (5.2)	35 (45.5)	0 (0)
Cruciferous vegetable^a^	77 (100)	36 (46.8)	8 (10.4)	31 (40.3)	2 (2.6)
Colored vegetable^a^	77 (100)	29 (37.7)	6 (7.8)	41 (53.2)	1 (1.3)
Milk^a^	51 (66.2)	9 (11.7)	20 (26.0)	16 (20.8)	32 (41.6)
Cheese^a^	68 (88.3)	6 (7.8)	24 (31.2)	21 (27.3)	26 (33.8)
Salt^b^	77 (100)	0 (0)	35 (45.5)	41 (53.2)	1 (1.3)
Sugar^b^	77 (100)	1 (1.3)	41 (53.2)	33 (42.9)	2 (2.6)
Sweetened condensed milk^b^	77 (100)	3 (3.9)	24 (31.2)	24 (31.2)	26 (33.8)
Seasoning^b ^	77 (100)	0 (0)	25 (32.5)	49 (63.6)	3 (3.9)
Coconut milk^b^	77 (100)	4 (5.2)	28 (36.4)	42 (54.5)	3 (3.9)
Cooking metho^d^					
Frying^b^	77 (100)	8 (10.4)	34 (44.2)	31 (40.3)	4 (5.2)
Stir frying^a^	77 (100)	8 (10.4)	16 (20.8)	53 (69.8)	0 (0)
Grilling(broiling)/	77 (100)	8 (10.4)	25 (32.5)	27 (35.1)	17 (22.1)
Barbecuing (charbroiling).^b^					
Roasting^a^	77 (100)	8 (10.4)	22 (28.6)	32 (41.6)	15 (19.5)
Boiling^a^	77 (100)	35 (45.5)	7 (9.1)	32 (41.6)	3 (3.9)
Steaming^a^	77 (100)	33 (42.9)	7 (9.1)	33 (42.9)	4 (5.2)

^a^Positive changes by increase intake; ^b^Positive changes by decreased or stop consumed

**Table 3:** Reasons and Types of Dietary Changes after a Cancer Diagnosis

Reasons for changes	Type of food
Increase the risk of cancer recurrence and reactivate the cancer cell.	Red meat, soy products, dairy products, sugar, certain types of fish
Side effects of treatment such as flatulence	Cruciferous vegetable and fruits that cause flatulence like jackfruit
Increased risk of non-communicable disease	Sugar, salt, sweetened condensed milk, seasoning, fries cooking method and use coconut milk.

**Table 4:** QoL and Correlation with Score in Dietary Changes (n=77)

Quality of life dimensions	Breast cancer n= 56	Gynecological cancer n= 21	p value^a^	All cancer (n=82) ^c^	r_s_	p value
GHS^a^	79.6±12.5	77.9±15.1	0.651	79.1±13.2	0.15	0.209
Functioning ^a^						
Physical	84.1±10.3	87.3±9.4	0.334	85.0±10.1	0.15	0.222
Role	72.0±28.1	77.5±30.2	0.408	73.5±28.6	-0.13	0.25
Emotional	84.7±18.6	87.9±17.2	0.496	85.6±18.0	0.27	0.024**
Cognitive	71.1±21.7	76.7±15.6	0.4	72,6±20.3	0.62	0.601
Social	94.3±13.8	100.0±0.0	0.051	95.9±12.0	0.12	0.298
Symptoms ^b^						
Fatigue	41.3±20.3	34.4±23.1	0.197	39.4±21.2	-0.28	0.017*
Nausea/ vomiting	31.4±16.8	20.0±21.3	0.024*	28.3±18.7	-0.07	0.519
Pain	31.4±16.8	20.0±21.3	0.024*	28.3±18.7	-0.07	0.519
Dyspnoea	11.3±23.5	10.0±19.0	0.927	10.1±22.3	-0.14	0.242
Insomnia	12.6±23.7	20.0±27.4	0.232	15.6±24.8	0.09	0.425
Appetite loss	11.9±25.4	3.3±10.3	0.194	9.6±22.5	-0.16	0.181
Constipation	11.3±21.6	10.0±21.9	0.725	10.9±21.5	0.27	0.823
Diarrhea	8.2±17.2	5.0±16.3	0.32	7.3±16.9	0.08	0.51
Financial difficulties	0.00±0.0	1.7± 7.5	0.104	0.4±3.9	-0.06	0.637

*p<0.05 significant using Man-Whitney U test; **p<0.05, significant correlation using Spearmen Rho test; ^a^ higher scale represents better functioning or healthy level; ^b^ higher scale represents a severe symptomatic problem.

## Discussion

This study revealed that dietary changes among Malay breast and gynaecological cancer survivors after cancer diagnosis. There was increased the intake of vegetables, however not fruits. In contrast to the studies by Shaharudin et al., (2013) and Yaw et al., (2014), the increased were reported for both fruits and vegetables after diagnosis of cancer. In the Malaysian eating culture, vegetables are always consumed as a complement to the main dishes hence this encourages increased consumption of vegetable rather than fruits. Vegetables are also widely available, cheaper and more accessible. Furthermore, misconception about reduced fruits intake was due to the high sugar content of the fruits and should be avoided (Dunn, 2018). There is still no clear evidence of the effect of high sugar intake from fruits on cancer as fruits are always appreciated for their properties of high fibre, vitamin and mineral (WCRF/AICR 2018b). 

Reducing sugar intake and stopping the consumption of sweetened condensed milk were positive changes made by the subjects in this study. Similarly, Velentzis et al., (2011) and dan Yaw et al., (2014) showed that cancer survivors have reduced their sugar intake after diagnosis of cancer. However, there is a misconception about the reduction of sugar intake due to the belief that sugar feed cancer and promote the development of cancer cell (Galsky, 2010). However, studies showed that consuming excess calories from sugar will increase the risk of obesity, which is one of the leading causes of cancer risk (Inoue-Choi et al., 2013, Fuchs et al., 2014). Sulaiman et al., (2014) found that an intake of 61g of sugar daily and equal to 14% of daily intake, increased the risk of breast cancer among pre and post-menopausal women. Besides, Vissers et al., (2015) found that cancer survivors with diabetes had lower QoL and more complications during treatments. 

Subjects in this study claimed that they stopped consuming milk and cheese after the diagnosis of cancer. Cancer survivors seem to believe that high protein foods will promote the growth of cancer cells (Yaw et al., 2014). Earlier studies have also shown that consuming dairy products increases the risk of ovarian and endometrial cancer and this was postulated to be due to the oestrogen and progesterone hormone content in milk (Ganmaa and Sato, 2005; Larsson et al., 2006). However, WCRF/AICR (2018c) reported there was limited evidence to conclude consumption dairy products and diets high in calcium in increasing the risk of the ovary and endometrium cancer. For breast cancer, the intake of dairy products may lower the risk of breast cancer among premenopausal women (WCRF/AICR 2018c). A meta-analysis by Wu et al., (2016) found that only consumption of skim milk decreased the risk of breast cancer by 4%, but not whole milk and total milk. Cancer treatment such as chemotherapy, removal of ovary, radiotherapy and menopause at an early age will increase the risk of osteoporosis among cancer survivors (Agrawal 2014; Campbell et al., 2019). Therefore, it is important to ensure adequate calcium intake among female cancer survivors. 

The results showed positive dietary changes had provided a better emotional function in subjects. A better socioeconomic status allowed subjects to make healthier food choices (Islami et al., 2017) and act as protective factors against depression (Freeman et al., 2016). This study finding is similar to studies by Mohammadi et al., (2013) and Wayne et al., (2006) healthy dietary changes led to better emotional states. Besides, a meta-analysis by Firth et al., (2019) also concludes that eating healthy food can reduce symptoms of stress. Healthy food intake provides vitamin, minerals and fibre which contains a variety of polyphenols and bioactive compound that help reduce stress and protect against mental illness (Firth et al., 2019).

Fatigue is a symptom most reported by cancer survivors due to the long-lasting neurotoxic effects from chemotherapy, anaemia, stress and menopausal symptoms (Asher and Myers, 2015). The potential relationship between dietary intake and fatigue symptoms weakens brain function leading to increased fatigue symptoms (Bitarafan et al., 2014). Also, healthy dietary changes can ensure an adequate intake of calories and nutrients to help reduce fatigue (Oh and Seo, 2011). The current study also found symptoms of fatigue were less reported by those who make positive dietary changes. This result was in line with the findings of the HEAL study which showed that improved nutritional quality led to a reduction in fatigue symptoms (George et al., 2011). 

It was noteworthy that the current study also found 31.2% of subjects were overweight and 33.5% were obese. Mean BMI in this study was higher than the studies by Shaharudin et al., (2013) and Yaw et al., (2014). Subjects in this study had longer duration of survivorship compared to Shaharudin et al., (2013). This explains by Vagenas et al., (2015) were increased in weight in line with the increase in survival. The number of overweight and obese groups increased from 57% to 68% during the 6-year follow-up period (Vagenas et al., 2015). Furthermore, gynaecological cancer survivors in this study had higher body weight and BMI compared to breast cancer and significantly affected overall BMI. The WCRF/AICR (2018a) recommends that cancer survivors achieve a healthy weight because obesity increases the risk of recurrent cancer and lowers the QoL.

There were several limitations identified in this study. This study involved only a small sample of sample size. However, this study was conducted over six months at two different hospitals which improved the subject’s socio-demographic diversity. The subjects selected for this study was part of a need assessment study for the development of education modules for cancer survivors. Besides, comparisons were not possible for types of gynaecological cancers due to the low incidence of uterine cancer (14.3%) compared with ovarian cancer (57.1%) and cervical cancer (28.6%). Furthermore, this study did not measure use the EORTC sub-modules specific to each type of cancer such as QLQ-BR 23, QLQ-OV28, QLQ-EN24 and QLQ CX-24 in assessing QoL. 

In conclusion, positive dietary changes have led to improved QoL in terms of emotional function and symptoms of fatigue. Dietary changes after cancer treatment were in line with the recommendations of the WCRF/AICR except for the intake of dairy products. Majority of breast and gynaecological cancer survivors were overweight and obese were found in this study. The low positive dietary change scores associated with obesity indicated that this group needs attention because they were at risk for recurrent cancer, non-communicable diseases and long-term side effects of treatment. Therefore, it is important to promote healthy dietary habits after cancer treatment that comes with weight management and overall health. 
